# Supplementary material for: The sustainability of recreational sports in Chinese cities based on cognitive entropy
Source: PLoS One. 2026 Jan 22;21(1):e0341012. doi: 10.1371/journal.pone.0341012 (PMC12826471; doi:10.1371/journal.pone.0341012)
Supplement: S1 Appendix — (DOCX) [file pone.0341012.s001.docx]

**Appendix:**

**Informed Consent Form**

**Research Title: Survey on Cognition and Satisfaction with Sports and Leisure Activities
Introduction:**You are invited to participate in a research study conducted by Chengdu Normal University. The purpose of this study is to understand urban residents’ awareness and satisfaction regarding sports and leisure activities, and to provide suggestions for promoting sustainable development and improving quality of life in cities.
**Procedures：**
If you agree to participate, you will be asked to complete a questionnaire that includes basic demographic information and questions about your awareness and satisfaction with various aspects of sports and leisure. The survey will take approximately 10–15 minutes to complete.
**Risks and Benefits:**
There are no foreseeable risks associated with this study. You will not benefit directly, but your participation may help improve urban sports and leisure services and policies.
**Confidentiality:**
Your responses will be anonymous and confidential. No personally identifiable information will be collected. Data will be used only for academic research and will not be shared with third parties.
**Voluntary Participation：**
Your participation is voluntary. You may refuse to participate or withdraw at any time without penalty.
**Contact Information:**
If you have any questions about this study, please contact:
Xuefang Zou
Tel: 18030642486/ Emai:369472397@qq.com
Chengdu Normal University
**Statement of Consent:**
By proceeding to complete the questionnaire, you indicate that you have read and understood the above information, and that you voluntarily agree to participate in this study.
