# Supplementary material for: The sustainability of recreational sports in Chinese cities based on cognitive entropy
Source: PLoS One. 2026 Jan 22;21(1):e0341012. doi: 10.1371/journal.pone.0341012 (PMC12826471; doi:10.1371/journal.pone.0341012)
Supplement: Attachment — Submitted filename: Response to the Reviewers.doc [file pone.0341012.s002.doc]

**Response to the** **referee’s comments**

Dear Dr. Xu,

Thank you very much for your letter and the referee’s comments on our manuscript (PONE-D-25-20453). The revisions are made according to the comments point by point, where the changes in the manuscript are highlighted with yellow background. 

**Reviewer 1**

1. **Comment：**Conceptualization of "Cognitive Entropy"

The theoretical underpinnings of "cognitive entropy" remain ambiguous. Authors should explicitly delineate its conceptual framework—distinguishing it from physical entropy—and justify its relevance to RS sustainability. Crucially, the derivation of the Stable Entropy (SE) formula necessitates rigorous mathematical grounding (e.g., Shannon entropy foundations) and validation of its adaptation to socio-ecological systems.

**Response:** Based on the findings against existing literature on urban RS sustainability, we have delineated the conceptual framework of our paper by distinguishing it from physical entropy, and justified its relevance to the RS sustainability. Especially, the series of entropy formulas have been derived and validated its adaptation to socio-ecological systems by using rigorous mathematical grounding, such as Shannon entropy foundations and the technique for order preference by similarity to an ideal solution (TOPSIS). Please see Section 4 (named 4. Evaluation of the RS system) of the revised version.

**2.** **Comment：**Methodological Transparency

Questionnaire Design: The rationale for employing 28 indicators across five subsystems lacks justification. Documentation of indicator loading matrices and psychometric properties (e.g., Cronbach’s α for reliability) should be provided in a supplementary table.

**Response:** Accepting the referee’s proposal, we have added the justification of the rationale for employing 28 indicators across five subsystems. By Employing advanced validation techniques (e.g., Structural Equation Modeling), the indicator loading matrices and psychometric properties (e.g., Cronbach’s α for reliability) have been provided. Please see Section 2 (named 2 Construction of the RS system) of the revised version.

**3. Comment：**Analytical Rigor

Entropy Computation: The assertion that "sub-dimension entropy equals the mean entropy of all items within that dimension" demands statistical justification—particularly regarding uniform indicator weighting and robustness against alternative aggregation methods.

**Response:** To eliminate the influence of its different items on the total entropy of the subsystem, the weights of a subsystem evaluation indexes are calculated by use of the TOPSIS method. Please see Section 4.2 and Table 5 (named 4.2 Sustainable development evaluation) of the revised version.

**4. Comment：**Interpretation of Findings

Causal Analysis: The identified lack of SD status in Cities remains unexplained. The study should identify causal drivers (e.g., weaknesses in specific subsystems like spatial infrastructure or experiential quality).

Generalizability: Claims about RS sustainability patterns must be contextualized within the study’s limitations (e.g., restricted to three cities), with caveats about extrapolation.

**Response:** (1) Causal Analysis —Accepting the reviewer’s suggestion, we have explained the SD status in the cities through weaknesses in specific subsystems like spatial infrastructure or experiential quality, and so on. Please see the Section 5(named 5. Suggestions for urban planning) of the revised version.

(2) Generalizability: Taking the referee’s advice, we have claimed about the RS sustainability pattern which has been contextualized within Chinese cities. Besides, we have added an acknowledgment of the study’s limitations and avenues for future research at the last of the paper. Please see the title of this paper and Section 7 (named 7.	Limitations and future research) of the revised version.

**Reviewer 2**

1. **Comment：**In the abstract ambiguity in describing how cognitive entropy theoretically connects with sustainability, unclear mention of “five subsystems” but later listing eight, inconsistent use of technical terms such as “sustainable entropy” without prior definition, and insufficient clarity in reporting empirical findings (e.g., Shanghai “not in SD state” is vague).

**Response:** In the abstract, we have redescribed how cognitive entropy theoretically connects with sustainability, and revised the listing subsystems, as well as avoided use some technical terms such as “sustainable entropy” without prior definition. At last, the reporting empirical findings have been clarified as much as possible by analyzing the calculation results of the research. Please see the abstract of the revised version.

2. **Comment：**In the introduction, issues include: a weak framing of the theoretical debate around sustainability of recreational sports, confusion between sustainability and development without deeper theoretical resolution, lack of integration of cited studies into a coherent research gap, and abrupt shifts in argumentation that reduce structural coherence.in order to increase the external validity of the study I am highly suggesting to cite the following articles: Comprehensive Evaluation of Urban Renewal Based on Entropy and TOPSIS Method; Developing Design Criteria for Sustainable Urban Parks; Socio-Psychological Effects of Urban Green Areas: Case of Kirklareli City Centre.

**Response:** (1) In the introduction, by following the expert’s advice, we have framed the theoretical debate around sustainability of recreational sports, clarified sustainability and development by integrating cited studies into the coherent research. Please see the instruction of the revised version.

(2) In order to increase the external validity of the study, we have accepted the reviewer’s suggestion and cited the pertinent literatures, such as Comprehensive evaluation of urban renewal based on entropy and TOPSIS method; Developing Design Criteria for Sustainable Urban Parks; Socio-Psychological Effects of Urban Green Areas: Case of Kirklareli City Centre. Please see the instruction and references of the revised version.

3. **Comment：**In the methodology, the issues include: unclear justification for sample selection in three cities, lack of theoretical explanation for linking entropy equations with subjective survey responses, inconsistent description of subsystems (sometimes five, sometimes eight), and a vague account of measurement where entropy is equated with cognition/satisfaction ratios without strong validation.

**Response:** (1) In the methodology, by accepting the expert suggestion, we have added the justification for sample selection in three cities. Please see Section 3.1 (named 3.1 Research context) of the revised version.

(2) We have theoretically explained the linkage of entropy equations with subjective survey responses, and derived the relationship between cognitive entropies and the sustainability of the RS by using rigorous mathematical grounding, such as Shannon entropy foundations and the technique for order preference by similarity to an ideal solution (TOPSIS). Please see Section 4 (named 4. Evaluation of the RS system) of the revised version.

4. **Comment：**In the discussion, problems include: overreliance on descriptive comparisons without deeper theoretical interpretation, weak connection to broader sustainability frameworks beyond entropy, and structural repetition of results instead of advancing argumentation.

**Response:** In the discussion, by following the expert’s suggestion, we have added theoretical interpretations of the results rather than structural repetition of results. Besides, we have added some suggestions for urban planning to enhance the sustainability of the RS system based on our results. Please see Section 5 (named 5. Suggestions for urban planning) of the revised version.

5. **Comment：**In the conclusion, the issues include: general statements without theoretical depth on how entropy advances urban sustainability research, over-simplification of findings without addressing limitations, absence of clear policy implications, and structural redundancy where the conclusion repeats earlier content rather than synthesising contributions.

**Response:** (1) In the conclusion, we have reorganized and theoretically explained how entropy advances urban sustainability research. Please see the Section 6 (named 5. Conclusions) of the revised version.

(2) The limitations of our findings have been added. Please see the Section 6 (named 7. Limitations and future research) of the revised version.

(3) Based the results of our research, we have made policy implications some advice about the sustainability of recreational sports for three cities by taking the suggestion of the expert. Please see the Section 5 (named 5 Suggestions for urban planning) of the revised version.

Looking forward to hearing from you soon.

Yours sincerely,

Xuefang Zou
